# Modeling Land Use Changes and their Impacts on Non-Point Source Pollution in a Southeast China Coastal Watershed

**DOI:** 10.3390/ijerph15081593

**Published:** 2018-07-27

**Authors:** Xin Zhang, Lin Zhou, Yuqi Liu

**Affiliations:** 1 State Key Laboratory of Remote Sensing Science, Institute of Remote Sensing and Digital Earth, Chinese Academy of Sciences, Beijing 100101, China; zhangxin@radi.ac.cn; 2 College of Remote Sensing Information Engineering, Wuhan University, Wuhan 430079, Hubei, China; 2015302590149@whu.edu.cn; 3 University of Chinese Academy of Sciences, Beijing 100049, China

**Keywords:** non-point source pollution, landscape pattern, geographic factors, HRULCI

## Abstract

Changes in landscape patterns in a river basin play a crucial role in the change on load of non-point source pollution. The spatial distribution of various land use types affects the transmission of non-point source pollutants on the basis of source-sink theory in landscape ecology. Jiulong River basin in southeast of China was selected as the study area in this paper. Aiming to analyze the correlation between changing landscape patterns and load of non-point source pollution in this area, traditional landscape metrics and the improved location-weighted landscape contrast index based on the minimum hydrological response unit (HRULCI) were applied in this study, in combination with remote sensing and geographic information system (GIS) technique. The results of the landscape metrics showed the enhanced fragmentation extent and the decreasing polymerization degree of the overall landscape in the watershed. High values of HRULCI were concentrated in cultivated land, while low HRULCI values mostly appeared in forestland, indicating that cultivated land substantially enhanced non-point source pollution, while forestland inhibited the pollution process.

## 1. Introduction

Water is the foundation for all life on earth. Water quality influences the environment greatly within a watershed ecological system [1]. A large amount of non-point source pollutants enter water-bodies through the process of runoff and groundwater migration, which are caused by human activities and urbanization and industrialization activities such as deforestation and excessive consumption of fertilizers. Water quality degradation threatens the whole ecosystem and human health [2].

According to the source of pollutants, we divide water pollution into two types: point source pollution and non-point source pollution [3]. The quantified monitoring of non-point source pollution is more difficult to conduct than point source pollution, due to the wide range of source of non-point source pollution. Hence, non-point source pollution becomes the dominant reason for water quality deterioration in watersheds gradually [4]. Non-point source pollution in river basins becomes more harmful mainly due to human activities, such as excessive fertilization and discharged livestock excrement. Landscape patterns have great influence on non-point source pollution from the perspective of pollutants transmission [5]. Non-point source pollutants transfer from their source to a river within the streams, and in this process, these pollutants go through various landscapes. Some landscapes can inhibit and absorb non-point source pollutants and can be categorized as sink landscapes, whereas other landscapes contribute substantially to pollutants and can be called source landscapes [6]. Consequently, various source-sink landscape patterns have different impacts on the load of non-point source pollution [7].

Remotely sensed data has gradually become a fundamental data source for landscape research due to the remarkable advantages of these data, as they are multitemporal, multiresolution and synchronous observations [8]. Jorgenson pointed out that the development of remote sensing techniques based on a growing array of satellite and airborne platforms that cover a wide range of spatial and temporal scales increasingly allows robust detection of landscape pattern changes [9]. Information about landscape patterns of a research area can be obtained accurately through the comprehensive use of multisensory remote sensing data [10]. Remote sensing techniques can be employed to study the impacts of landscape patterns on the non-point source pollution loads [11].

According to the classification standard of land use and non-point source pollution, combined with the practical situation of Jiulong River basin, we drafted a classification scheme of the source-sink landscape and analyzed the dynamic changes in the source-sink landscape after extracting the land use information of multitemporal landscape patterns of the study area [12]. Various landscape metrics, such as Largest Patch Index (LPI), Landscape Shape Index (LSI), Mean Nearest Neighbor Distance (ENN_MN), Interspersion and Juxtaposition Index (IJI), Area Weighted Mean Shape Index (AWMSI), Number of Patches (NP), Patch Density (PD) and Aggregation Index (AI), were calculated to analyze the change in spatial structure in Jiulong River basin during the years of the study. However, these metrics characterized the spatial distribution and fragmentation degree of the landscape and they were not of ecological significance, thus an additional integrated research method was needed to analyze the impact of landscape patterns on specific ecological processes.

A location-weighted landscape contrast index (LCI) based on the theory of “pattern and process” which was mainly focused on the correlation between landscape pattern and certain ecological process in landscape ecology was proposed to evaluate the effect of landscape pattern on non-point source pollution loads [13]. LCI is scale independent and it can characterize the relative contribution of landscape pattern to a specific monitoring point, and the value is positively correlated with the contribution. Based on the spatial load contrast index, Jiang put forward grid landscape contrast index (GLCI) which reflected the contribution of landscape in each grid to non-point source pollution [14]. However, in the former studies, only surface distance, slope and relative height were taken into account in the calculation of LCI, which was obviously not comprehensive. An improved LCI model was constructed, taking more geographical factors into account, such as soil moisture, soil texture, land use types and annual precipitation [15]. The hydrological response unit (HRU) with a single land use and soil type was applied as the smallest study unit in this paper. The location-weighted landscape contrast index was computed on the basis of the minimum hydrological response unit in this study (HRULCI). The impact of land use change on non-point source pollution was analyzed through the integrated analysis of landscape-pattern changes and multitemporal HRULCI values [16]. The objective of this paper is to analyze the correlation between dynamic spatial distribution of landscape and change on load of non-point source pollution. The calculation results can be applied in the manipulation of future landscape pattern for the sustainable development in Jiulong River basin.

## 2. Study Areas and Data Sources

**Figure 1:**
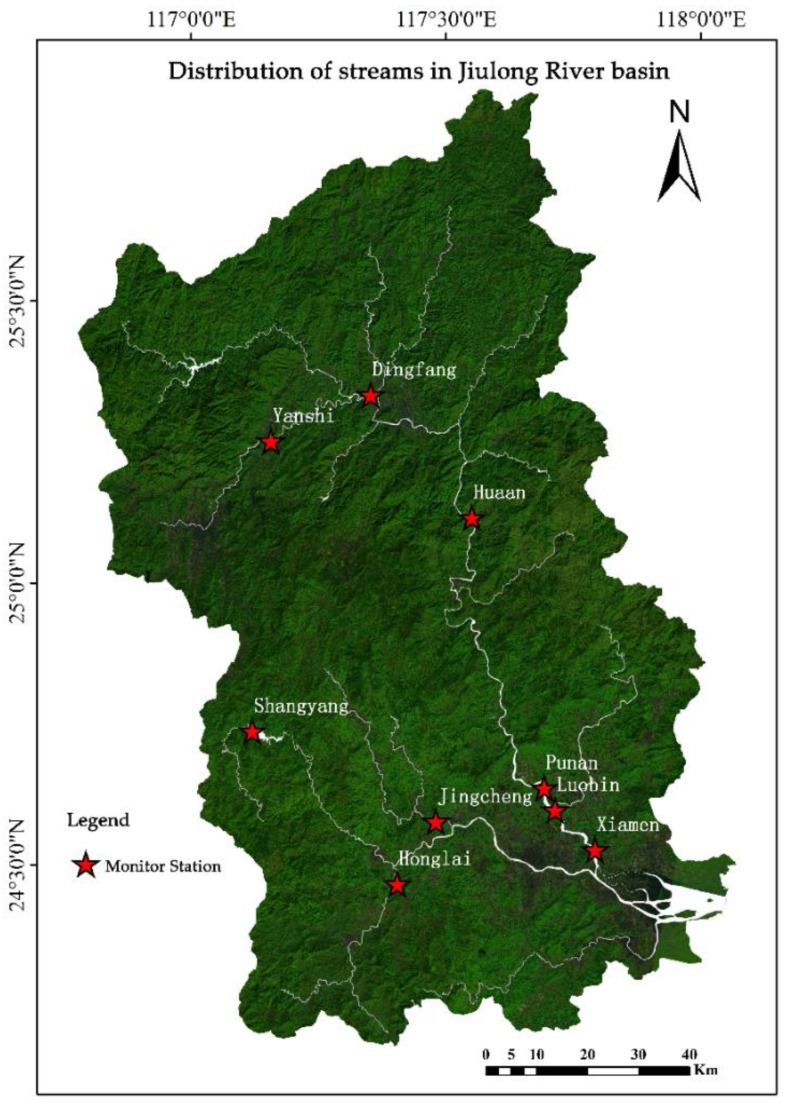
The specific situation of Jiulong River basin.

Jiulong River basin located in southeast of China was selected as the research area. This basin covers an area of 14,745 km^2^ bounded between 116°47′ E to 118°02′ E and 24°13′ N to 25°51′ N [17]. Landscape patterns have changed recently due to the urbanization and industrialization during the study period (from 2005 to 2017). Vegetative cover has been damaged from overexploitation of resources, such as the exploration of mineral resources and the rapid increase of cultivated land [18], resulting in severe soil erosion in some areas [19]. Animal husbandry has been developed in the drainage area, and the excrements of livestock cause serious water pollution. Construction of many hydropower stations without considering the ecological capacity of Jiulong River affects the water purification capacity of the river [20]. Many factors have led to the deterioration of the river from non-point source pollution, and water quality degradation has an impact on production activities in Jiulong River basin [15]. Figure 1 displayed the coverage of the Jiulong River basin.

Landsat TM/OLI remote sensing images were selected as the fundamental data sets in this study [21], and Table 1 displays the time when the images were acquired. A digital elevation model (DEM) of this area with a resolution of 30 m was downloaded from ASTER GDEM [8]. Soil type data were 1:1,000,000 and provided by the Institute of Soil Science for the Second National Land Investigation, and the main soil classification system was FAO-90. Annual average precipitation data were obtained from the National Meteorological Data Sharing Platform. The consumption data of fertilizers were acquired from statistical yearbooks of different years for Fujian Province.

## 3. Methods

### 3.1. Source-Sink Landscape Classification

**Figure 2:**
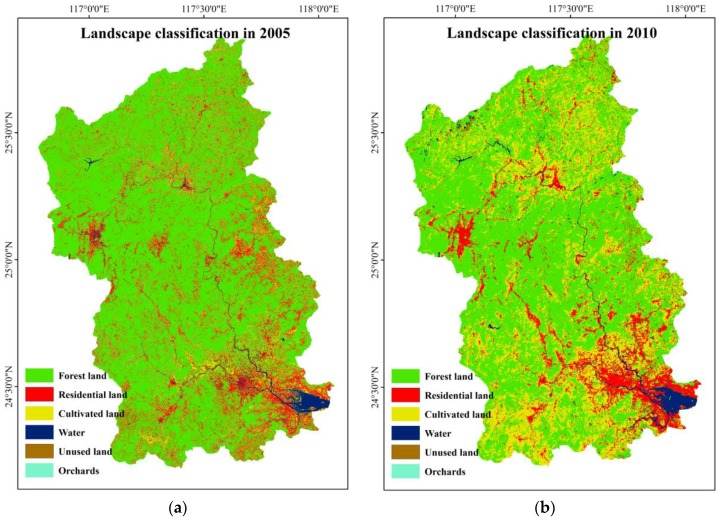

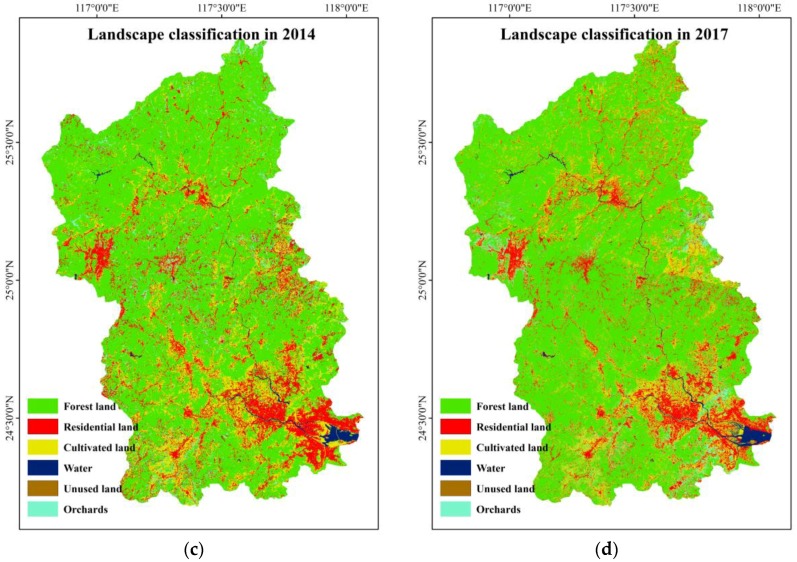
Classification results of multitemporal remote sensing images in Jiulong River basin. (**a**) Landscape classification in 2005; (**b**) Landscape classification in 2010; (**c**) Landscape classification in 2014; (**d**) Landscape classification in 2017.

According to the national land use classification standards, combined with the various land use types referenced in the secondary classification standard, the study area was categorized into six types, viz, forestland, residential land, cultivated land, water, unused land and orchards. The supervised classifier was applied in this paper, and the land use classification results of 2005, 2010, 2014 and 2017 are represented in Figure 2.

According to the classification results, we computed the area of each landscape of Jiulong River basin in 2005, 2010, 2014 and 2017. The land use change of Jiulong River basin was displayed in Table 2.

The specific values of the interpretation accuracy are displayed in Table 3. The overall classification accuracy is higher than 80%, and kappa indexes are larger than 0.83.

On the basis of source-sink theory in landscape ecology, source landscapes contribute non-point source pollutants and accelerate the increase on load of pollution, and in the contrast, sink landscapes can intercept or absorb the polluting substances [14]. A watershed ecosystem will be sustainable only when the migration between source and sink landscapes remains balanced. Source-sink landscapes with various compositions and spatial distributions have different impacts on non-point source pollution. Hence, based on the premise that various source-sink landscapes affect non-point source pollution greatly and according to the source-sink theory, we categorize residential land, cultivated land and orchards as source landscapes, and forestland, water and unused land are considered sink landscapes in this paper.

### 3.2. Calculation of Landscape Metrics

Landscape indexes can reflect the spatial structures of landscape patches, such as patch size, shape, arrangement, connectivity and adjacency. The selected landscape metrics should be weakly correlated and characterize the landscape pattern comprehensively. Eight landscape metrics were applied to study the landscape patterns in Jiulong River basin. Table 4 contains specific information on the eight metrics [22,23,24].

### 3.3. HRULCI

Landscape metrics cannot characterize the spatial heterogeneity of a landscape as well as traditional remote sensing indexes such as normalized difference vegetation index (NDVI). More importantly, existing landscape metrics commonly analyze shape, distribution and aggregation of landscape patches and use an independent computing procedure associated with a certain ecological process. Based on the drawbacks, a hydrological response unit landscape contrast index (HRULCI) was calculated in this paper.

#### 3.3.1. Division of HRU

ArcGIS was applied to divide the watershed into multiple HRUs with only a single land use type and a soil type depending on the DEM data with the resolution of 30 m, land use classification data and soil type data [25,26]. The DEM data were acquired from ASTER GDEM, and the soil type datasets were obtained from the Institute of Soil Science for the Second National Land Investigation. In addition, the spatial distribution characteristics of the underlying surface factors were represented in a discretized form. It is crucial to extract the drainage system from DEM efficiently and accurately. We set different thresholds for the extraction and compared the extracted drainage water system with the actual situation to determine the final threshold in the hydrological module of the software, and we obtained the rational distribution of the drainage in Jiulong River basin. HRUs of the watershed were determined through an overlay analysis applied to sub-watershed data and landscape type information. The threshold of area was defined according to the actual situation, when the area of a HRU was smaller than the threshold, we categorized it into HRUs with small area. HRUs with small areas were merged into other HRUs.

**Figure 3:**
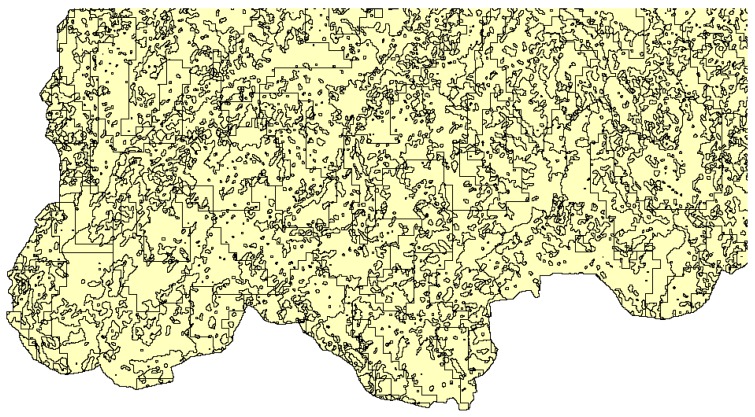
A part of the hydrological response unit (HRU) division results of Jiulong River basin in 2010.

The division results of the HRUs for Jiulong River basin in 2010 are displayed in Figure 3 and 693 HRUs were obtained in this study. Calculating the indexes based on HRUs not only presents the spatial differences in the underlying surface but also reflects the internal uniformity of each study unit in terms of land use and soil type.

#### 3.3.2. Calculation of Correction Coefficient

Effective soil moisture, soil texture, slope, land use types, annual precipitation, the effective distance and NDVI were selected as the geographical factors for the index calculation in this paper.

Effective soil moisture and soil texture represents the soil properties of the watershed, mainly reflecting the impacts of soil on surface runoff. Slope reflects the terrain characteristics of this area. Land-use types are the foundation of distinguishing the source-sink landscape, and different source (sink) landscapes differ based on the influence of the transmission of non-point source pollutants. Annual precipitation is a crucial indicator of climate in the watershed of the study. The effective distance refers to the farthest distance that non-point source pollutants affect the water body. In general, the closer the non-point source output is from the water, the more obvious the diffusion effect of the pollutant is. NDVI represents the information of surface vegetation effectively, and an area with a higher NDVI possesses a larger amount of vegetation and a stronger capacity to prevent the transmission of non-point source pollutants.

**Figure 4:**
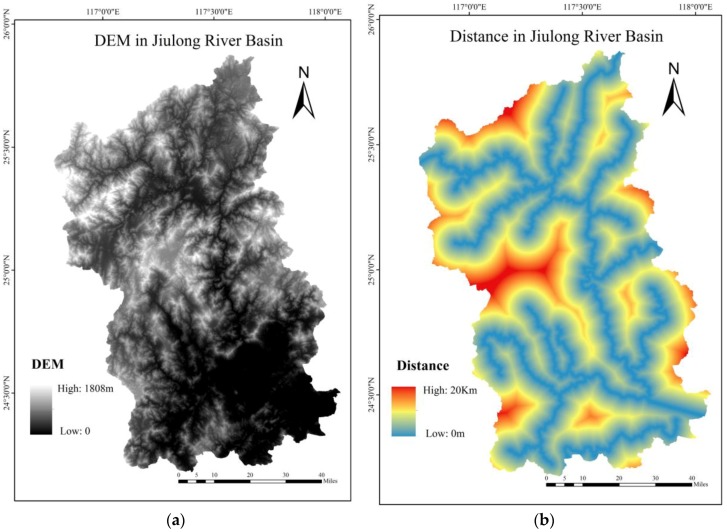

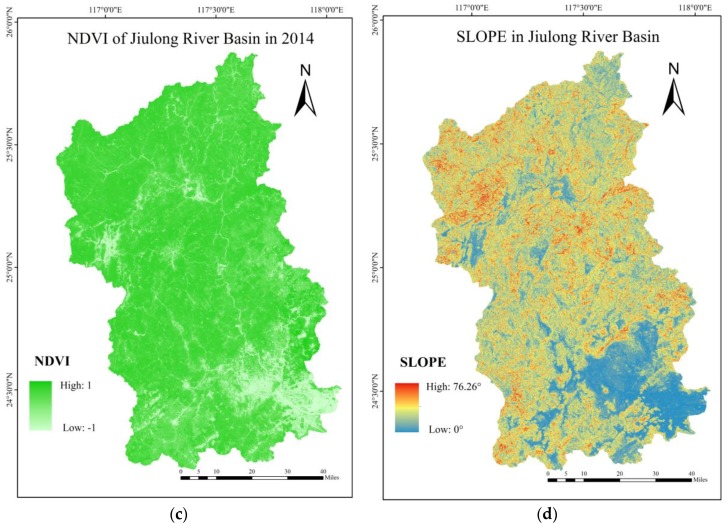
The geographical factors for the index calculation. (**a**) digital elevation model (DEM) in Jiulong River basin; (**b**) The effective distance in 2017; (**c**) normalized difference vegetation index (NDVI) in 2014; (**d**) Slope data of Jiulong River basin.

DEM, slope data, and NDVI in 2014 and the effective distance in 2017 of Jiulong River basin are presented in Figure 4.

Based on the Chinese standard cultivated land runoff pollutant discharge coefficient and according to the actual situation of Jiulong River basin in 2005, 2010, 2014 and 2017, the coefficients of the geographical factors were corrected. We referred to the correction standards of the geographic factors proposed in a former study [15].

#### 3.3.3. Calculation of HRULCI

Landscape metrics that are selected to study the effect of landscape patterns on water quality must be of ecological significance. The improved landscape indices should reflect the correlation between landscape pattern and the non-point source pollution transmission. The HRULCI, which describes the effect of a source-sink landscape on the transmission of non-point source pollutants from generating plots to a water body, was applied in this study.

According to the calculation process of the location-weighted landscape contrast index proposed in an earlier study [15], HRULCI was computed. According to the emission coefficient of standard farmland in China, three types of non-point source pollutants (N, P, COD) are used to measure the relationship between landscape pattern and non-point source pollution, and the specific calculation formulas are expressed as follows:

The computing criterion of location-weighted landscape contrast index is presented as:

(1) $${\mathrm{HRULCI}}_{i}=W_{i}\times A_{i}\;$$

where *i* refers to a certain HRU, *A_i_* represents area of the HRU, *W_i_* is the generation/inhibition weight of the HRU for non-point source pollutants. *W_i_* is influenced by land use, soil properties, precipitation and fertilizer application. In addition, it is important to correct various geographical factors given the process of non-point source pollutants transferring through HRUs to a water body, and we express *W_i_* as:

(2) $$W_{i}=f\left(L,P,R,D,N,S,F,A\right)\;$$

where L, P, R, D, N, S, F, A are correction coefficients of different source-sink landscapes, slope, annual precipitation, distance elements, NDVI, soil type, fertilizer application amount and effective soil moisture respectively. According to the emission coefficient of standard farmland (the characteristics of the so-called standard farmland are as follows: the area is the plain, the crop is wheat, the soil type is loam, the fertilizer application amount is 375–525 kg/ha/year, the precipitation is within the range of 400–800 mm) in China, the basis of the amendment of relative parameters can be seen in Table 5.

Standardization of these correction coefficients is necessary to compare the location-weighted landscape contrast index of different HRUs. The normalization formula is expressed as

(3) $$\;W=\frac{W_{i}}{W_{max}}\;$$

where *W_i_* is the non-point source pollutants correction coefficient of a certain landscape, *W_max_* is the maximum value of non-point source pollutants correction coefficient of a certain landscape.

If there are different kinds of non-point source pollutants, we have the expression as

(4) $$\;{\mathrm{HRULCI}}_{XY}={\mathrm{HRULCI}}_{X}+{\mathrm{HRULCI}}_{Y}\;$$

where HRULCI*_Y_* refers to the location-weighted landscape contrast index of pollutant *Y* in HRU, HRULCI*_XY_* equals to the sum of *X* and *Y*.

As for the sub-basin, the formula of location-weighted landscape contrast index of non-point source pollution is displayed as

(5) $$\;{\mathrm{HRULCI}}_{sub}=\sum_{i=1}^{N}m{\mathrm{HRULCI}}_{i}\;$$

where *N* is the total amount of HRU*_s_* in the sub-basin, *i* refers to a specific HRU. Comparing the HRULCI*_sub_* values of various sub-basins, the higher value means greater spread of non-point source pollution in the sub-basin and a higher probability of nutrient loss in this HRU.

## 4. Results and Discussion

### 4.1. Land Use Change Analysis

**Figure 5:**
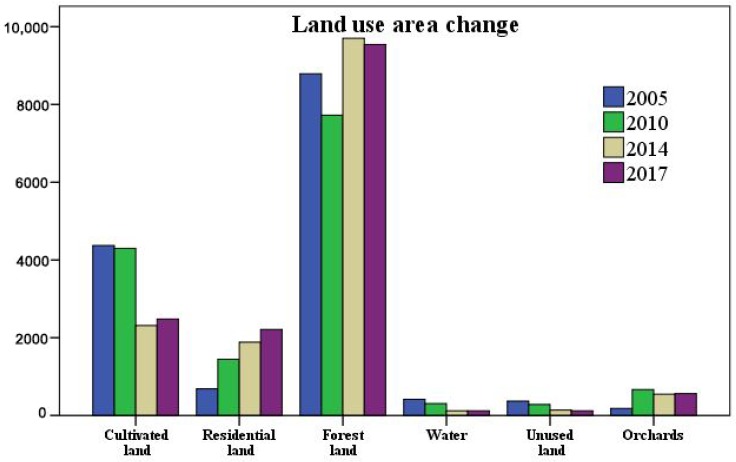
Change in land use area from 2005 to 2017.

Land use area changes during the experimental period are displayed in Figure 5. Comparing the area of the various landscapes in different years, we made a few basic conclusions.

The area of forestland increased slightly, but the growth was not obvious given its large area. Cultivated land showed a decreasing trend, especially from 2010 to 2014, and its area decreased rapidly, which could be result of effective implementation of the Grain to Green policy in Jiulong River basin [27]. The area of water showed a significant decreasing trend during the study, which indicated a decrease in water resources in Jiulong River basin. While extracting the distribution of the water system in Jiulong River basin, the drainage system contracted, which was due to the narrowing of part of the river channels and the reduction of reservoir areas.

The area of residential land grew obviously from 2005 to 2017, indicating further urbanization in Jiulong River basin. We inferred that the main reason for the increase in area increase residential land was the increase in residential land area.

The area of orchards decreased slightly after a rapid increase during 2005–2010. The reduction was caused by the orchards to forestland policy adopted in Jiulong River basin. In some areas with a severe loss of water and soil, this reduction was particularly obvious.

The area of unused land decreased throughout the study period, which indicated an increasing extent of land use in Jiulong River basin. The destruction of natural landscapes such as forestland and water in the earlier stages of human activities led to a large number of unused land. However, with the improvements in human activities and the enhancement value of land, unused land has been utilized gradually and has been transferred to other land use types.

### 4.2. Landscape Metrics Results

The calculated landscape indexes are displayed in Table 6.

On the basis of Table 6, NP showed an obvious increasing trend from 2010 to 2017, after a decrease during 2005–2010. PD presented a similar trend, and both NP and PD increased overall. We inferred that this phenomenon was a consequence of the enhanced landscape fragmentation degree caused by the human activities in Jiulong River basin.

LPI remained stable during 2005–2017, which indicated that the abundance of the dominant landscape, forestland, was not substantially disturbed. Human activities had minimal impacts on forestland, and the area of forestland remained stable in the study years.

LSI characterized the shape of the landscape, and a higher LSI indicated a more complicated landscape shape [12]. LSI decreased during 2005–2014 and then increased rapidly during 2014–2017, and in 2017, LSI was much larger than it was in 2005. The change trend showed that the landscape pattern tended to be complex after a trend of being simple. Analyzing this trend combined with land use changes, this phenomenon was due to the rapid increase in residential land area during 2014–2017, which destroyed the original landscape pattern.

AWMSI had a trend similar to LSI. The increase in LSI reflected the complexity of landscape shape, while the increasing AWMSI indicated a more enhanced edge effect of the landscape [28]. Enhanced edge effects made pollutants transmission among various source-sink landscapes easier.

ENN_MN had change characteristics similar to IJI, which increased first and then decreased. ENN_MN and IJI reflect the neighboring distance and distribution of the same landscape type, respectively. ENN_MN and IJI measure the distribution of adjacencies among patch types. The trend of decreasing after increasing showed the concentrated tendency within the same landscape type [23]. We speculated that the trend was due to the significant increase in forestland and residential land after 2010 with a combination of land use change information, and the increased area of the dominant landscape, forestland, led to a decrease in the index, while the expansion of residential land was based on the existing residential land. Hence, ENN_MN and IJI decreased in residential land as well.

AI which was class specific measured the aggregation in landscape pattern [29]. He stated that AI was the quantitative basis to correlate spatial patterns with the process which were class specific [30]. AI presented a slow upward trend during 2005–2010 but fell rapidly after 2014, indicating a decreasing overall landscape polymerization degree. This result was consistent with those for NP and PD, and the extent of landscape fragmentation increased, while the degree of landscape polymerization decreased.

### 4.3. Analysis of HRULCI Changes

**Figure 6:**
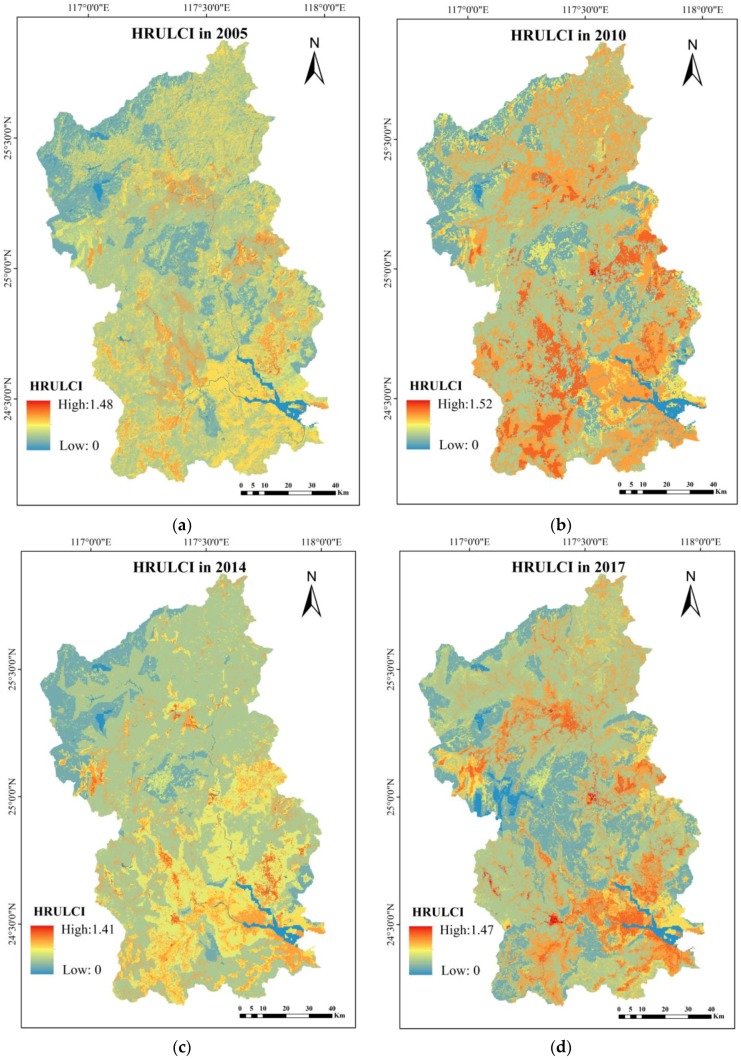
Location-weighted landscape contrast index computed on the basis of the minimum hydrological response unit (HRULCI) calculation results in Jiulong River basin. (**a**) HRULCI in 2005; (**b**) HRULCI in 2010; (**c**) HRULCI in 2014; (**d**) HRULCI in 2017.

The results of the HRULCI of Jiulong River basin in 2005, 2010, 2014 and 2017 are shown in Figure 6.

The results of HRULCI show that numerically higher areas were concentrated in cultivated land use because cultivated land was covered with less vegetation, and soil texture was loose; therefore, soil erosion and nutrient loss on the cultivated land were more severe [13]. Additionally, the application of fertilizers resulted in cultivated land playing an important role as a source of non-point source pollution.

The mid-range values were concentrated in residential land and orchards. Residential land with a low vegetation cover ratio was mostly impervious to water. Soil erosion and nutrients were not obvious in residential land, but the garbage produced by residents contributed substantial non-point source pollutants. Hence, the HRULCI of residential land was second only to that of cultivated land. Compared with soil on cultivated land, soil in orchards was more compact, and the soil and water conservation function of fruit trees was stronger than crops, yielding a smaller HRULCI value in orchards than in cultivated lands. HRULCIs in the above two land use types were higher than 1, indicating that orchards and cultivated land enabled the transmission of non-point source pollutants.

Chen pointed the view that a smaller LCI represents greater inhibition capacity [13]. Values of HRULCI in forestland were lowest because of the high vegetation cover rate and strong capacity to conserve water and soil. HRULCI was lower than 1, indicating that forestland inhibited the transmission of non-point source pollution.

### 4.4. Discussion

Compared to the traditional non-point source pollution model (SWAT, AGNPS, GLEAMS, etc.), this paper evaluated the relationship between landscape pattern and non-point source pollution from another perspective. The calculation result of the traditional non-point source pollution model is always a fixed value, which was used to estimate the non-point source pollution load in the study area [31,32,33]. But there are some disadvantages in these methods, specifically, a numerical result can not reflect the spatial difference of non-point source pollution load inside the study area. For example, in the same study area, the non-point source pollution load may be similar in different years, but the internal landscape pattern may have been greatly changed, and the traditional non-point source pollution model calculation results can not reflect this phenomenon. Based on the above reasons, this paper changes the quantitative calculation method of the nonpoint source pollution load, and evaluates the relationship between the different source and sink patterns on the transmission process of non-point source pollutants. Compared with the traditional non-point source model, the proposed method can be simpler to show the influence of different landscape regions on the transport of non-point source pollutants in the study area, and the calculation results can effectively show the spatial difference of the non-point source pollution in different regions of the study area.

The analysis of changes of land use types is not associated with the ecological processes, and this method is insufficient to measure the effects of landscape pattern on load of non-point source pollution in this paper. HRU with a single land use and soil type was taken as the minimum research scale in this study, HRULCI was thus of ecological significance. Jiang established GLCI to reflect the contribution of landscape in a certain space to non-point source pollution [14]. Compared to the artificial division grid with a fixed size, the land use and soil types are homogeneity in the same HRU. The landscape pattern was related with ecological processes effecting non-point source pollution by constructing HRUs. When researchers take the grid unit as the minimum research scale, the scale effect should be noted and it should be verified whether the grid unit is appropriate for the research. While the use of minimum HRU as the minimum research scale can avoid the uncertainty of research scale.

## 5. Conclusions

In this paper, we analyzed the change of land use portions in Jiulong River basin and calculated eight traditional landscape metrics in 2005, 2010, 2014 and 2017. The area of residential land and orchards increased rapidly, while unused land and water decreased, which reflected the imbalanced migration between source and sink landscapes. Landscape metrics quantify and characterize spatial structure of landscape pattern. These metrics reflect the fragmentation extent and configuration features of the study area, and explain some of the variation in water quality not explained by land use portions [8], while they are not linked with specific ecological process [34]. It was concluded from the calculation results of landscape metrics that the overall landscape pattern of Jiulong River basin tended to be more fragmented.

An HRULCI with ecological significance and multiscale suitability was applied to qualitatively analyze the different effects of various landscapes on the transmission of non-point source pollutants. HRU with a single land use and soil type was used as the minimum unit for the calculation of the improved location-weighted landscape contrast index in this paper. The calculated results of HRULCI are important for two reasons. On the one hand, areas with a high HRULCI should be the key management areas, and decreases in the HRULCIs in such areas will be beneficial for controlling non-point source pollution. On the other hand, changes in a source-sink landscape within a watershed can be obtained by analyzing changes in the HRULCI over a certain time gradient. Increases in HRULCI values indicate the dominant status of source landscape in a drainage area and vice versa. Analyzing the HRULCI in a watershed is the foundation for studying source-sink landscape changes and predicting the change trend of a landscape in a river basin.

## Figures and Tables

**Table 1:** Multitemporal images and sensors information table.

Images Acquisition Time	Sensor Type
2017-10-02	Landsat8 OLI
2017-10-25	Landsat8 OLI
2017-08-06	Landsat8 OLI
2014-09-08	Landsat8 OLI
2014-10-17	Landsat8 OLI
2014-10-17	Landsat8 OLI
2010-05-24	Landsat5 TM
2010-10-03	Landsat5 TM
2010-10-19	Landsat5 TM
2005-11-05	Landsat5 TM
2005-10-05	Landsat5 TM
2005-10-05	Landsat5 TM

**Table 2:** The percent of land use change of Jiulong River basin from 2005 to 2017 (km^2^).

Year	2005	2010	2014	2017
Forestland	59.33%	52.48%	65.98%	63.46%
Residential land	4.63%	9.82%	12.83%	14.71%
Cultivated land	29.51%	29.21%	15.73%	16.50%
Water	2.81%	2.07%	0.81%	0.79%
Unused land	2.50%	1.91%	0.94%	0.81%
Orchard	1.22%	4.51%	3.71%	3.73%

**Table 3:** Classification accuracy table.

Year	2005	2010	2014	2017
Overall accuracy	86.20%	89.45%	90.12%	88.34%
Kappa	0.83	0.86	0.87	0.85

**Table 4:** The specific descriptions for eight landscape metrics.

Landscape Metrics	Description
Largest Patch Index (LPI)	LPI indicates the share of the landscape that is occupied by the largest patch of the landscape.
Landscape Shape Index (LSI)	The sum of all patch perimeters is divided by an amount equivalent to the perimeter of a circle with the same area as the landscape area to calculate LSI.
Mean Nearest Neighbor Distance (ENN_MN)	ENN is calculated only if at least two patches of a corresponding type occur. ENN characterizes the landscape partially.
Interspersion and Juxtaposition Index (IJI)	IJI is calculated from the relationship between the length of each edge type and total edge of the landscape, divided by a term based on the number of landscape types.
Area Weighted Mean Shape Index (AWMSI)	AWMSI is computed by weighting patches according to their size.
Number of Patches (NP)	The number of patches in the landscape under investigation is counted.
Patch Density (PD)	The number of patches per unit area in the landscape.
Aggregation Index (AI)	AI indicates the degree of patch clustering, ranging from 0 to 100.

**Table 5:** The relative coefficient calculation methods table.

Relative Parameters	Basis of Amendment
Landscape (L)	It is divided into three types: forest land, cultivated land and orchard, and other land use types.
Slope (P)	For reference standard farmland, the slope is divided into two degrees: below 25 degrees and above 25 degrees.
Annual precipitation (R)	It was divided into three categories: below 400 mm, 400 mm to 800 mm, above 800 mm.
Distance (D)	Choose the 20 km as the maximum surface distance of non-point source pollution from area to local water conservation.
NDVI (N)	It is used to reflect the biomass information of the vegetation.
Soil type(S)	The classification standard is the proportion of sandy soil, loam and clay.
Fertilizer application (F)	It was divided into three categories: below 25 kg, 25 kg to 35 kg, above 35 kg.
Effective soil moisture (A)	It was used to measure the capacity of soil and water conservation in different regions of the basin and the ability to generate surface runoff under the same conditions.

**Table 6:** Landscape index calculation results table.

Landscape Indexes	2005	2010	2014	2017
NP	156,570	121,732	129,869	163,751
PD	6.4292	4.1052	5.3498	11.8081
LPI	5.2191	5.2414	5.2439	5.55
LSI	206.7832	172.9367	161.2178	236.6683
AWMSI	25.2509	17.6869	18.7862	29.2333
ENN_MN	318.9282	360.7935	319.132	299.6323
IJI	20.8337	24.6556	24.114	20.1225
AI	73.6442	77.9723	79.4614	62.3321
